# A Defense of The-Risks-of-Daily-Life

**DOI:** 10.1353/ken.2017.0033

**Published:** 2017-09

**Authors:** Ariella Binik

## Abstract

Research examining the safe and effective treatment of diseases and disorders affecting children offers one of the best prospects for improving the medical treatment of children. But the inclusion of children in research raises difficult ethical questions, among them: To how much risk is it permissible to expose children in research? Various thresholds have been proposed to constrain research risks that do not offer children the prospect of direct medical benefit. These proposals include limiting research risks to (1) the risks of routine medical examinations, (2) the risks of participation in charitable activities, (3) the risks of family life, and (4) the risks-of-daily-life. I examine which, if any, of these proposals is defensible. I argue that only the risks-of-daily-life threshold is defensible and I offer a new justification for this risk threshold. I argue that the risks of daily life are justifiable because they are part of a reasonable trade-off between personal safety and our ability to pursue meaningful lives.

Most agree that clinical research offers one of the best prospects of improving pediatric medicine. Most also agree that children may be exposed to some degree of risk while participating in clinical trials. But the degree of risk that should be permitted and the reasons for which it should be permitted remain controversial. In this paper, I examine a central risk threshold in research with children—the threshold constraining risks that do not offer research subjects the prospect of direct medical benefit. That is, the threshold for research risks undertaken in the interests of deriving valuable knowledge that may benefit future children, but not the research subjects themselves. I examine competing proposals for this risk threshold and offer a new defense for an existing threshold.

Commentators have proposed a variety of different thresholds to constrain risks without benefit in research with children. These proposals include limiting risk according to (1) the risks of routine medical examinations (CIOMS 2002; Kopelman 2004), (2) the risks of [Other P-413]participating in charitable activities (Wendler 2005; 2010), (3) the risks of family life (Ackerman 1980; Nelson and Ross 2005) or (4) the risks-of-daily-life (DHHS Regulations 1982; Freedman, Fuks, and Weijer 1993). Of these, I argue that only the risks-of-daily-life threshold is defensible, but not for the reasons usually offered. That is, I raise a novel objection to the current justification of the risks-of-daily-life threshold and then I propose a new justification. I argue that daily risks are morally relevant because they are part of a reasonable trade-off between personal safety and our ability to pursue meaningful lives. I then argue that this new defense of the risks-of-daily-life threshold is more persuasive than its competitors. This work aims to help clarify the role of risk assessment within the ethical justification for research with children. It also aims to identify a risk threshold that offers adequate protections for children without unduly restricting valuable research.

I proceed as follows: First, I explain the moral problem complicating children’s participation in research. Second, I outline necessary components of a successful solution, including the need for a risk threshold. Third, I argue that three of the most prominent proposals for risk thresholds fail to meet the necessary criteria. Fourth, I examine the risks-of-daily-life threshold. I draw attention to a problem with the most prominent justification of this threshold. I then propose a new argument justifying the risks-of-daily-life. Finally, I argue that this proposal is more successful than its rivals in justifying risk and potential benefit judgments for clinical research.

## 1. BACKGROUND: THE JUSTIFICATION FOR PEDIATRIC RESEARCH

When competent adults participate in research, their exposure to risk is justified, at least in part, by the principle of informed consent. Informed consent helps to uphold the moral principle of respect for persons and for their autonomous decisions to enroll in a trial. But when a research subject—such as a child—cannot provide informed consent, it is not clear what (if anything) justifies her participation in research.

Ethicists have proposed a number of different reasons why children’s inclusion in clinical research may be permissible in the absence of informed consent. Some argue that children’s participation is justifiable because research offers long-term benefits for the medical treatment of children as a group (Shirkey 1968; Levine 1986; McCormick 1974; Spriggs and Caldwell 2011). Others argue that informed consent is not a necessary ethical requirement. In certain circumstances, surrogate consent from [Other P-414]parents or guardians may justify children’s inclusion (Ross 1998; Freedman 1975; Levine 1986; Ackerman 1979). Still others claim that it is permissible to enrol children when a research protocol’s risks stand in reasonable relation to the knowledge to be gained. That is, the risks of a trial must be offset by the potential benefits of the research (Weijer 2000; Freedman, Fuks, and Weijer 1992; Miller and Weijer 2006).

Each of these arguments is a necessary part of the ethical justification for pediatric research, but more needs to be said about how to determine when the risks of a trial stand in reasonable relation to the knowledge to be gained. In particular, more needs to be said about permissible risks for vulnerable populations. When a research protocol proposes to include a vulnerable population, such as children, an upper limit is invoked to constrain risks that do not offer medical benefit. This threshold is often called the minimal risk threshold.^1^ The minimal risk threshold functions as an additional requirement over and above ethical requirements governing the inclusion of competent adults in research. Accordingly, the minimal risk threshold plays a central role in the protection of children in research. But this risk threshold is inadequately justified. One difficult question raised by the minimal risk threshold is: how much risk should be permissible and why is this amount of risk permissible? In other words, how should minimal risk be defined? In the following, I consider four of the most prominent interpretations of the minimal risk threshold. I defend the idea that minimal risk should be understood as the risks-of-daily-life and I propose a new justification for this risk threshold.^2^

## 2. CRITERIA FOR A SUCCESSFUL RISK THRESHOLD

To evaluate the success of competing interpretations of minimal risk, it is useful to first consider the necessary criteria for a successful interpretation. There are at least three criteria: First, an interpretation should meet the requirement of *generality*—this is the idea that a successful interpretation must apply to children of all ages (e.g., infants through nonautonomous teenagers). This criterion helps to ensure that no group of children is excluded from research without justification. The requirement of generality also aims to ensure that a proposed risk threshold will recognize differences between children of different ages and will offer ways to accommodate these differences.

Second, a successful interpretation should meet the requirement of *fidelity*. That is, it must be consistent with common moral intuitions about the permissible treatment of children (such as the intuition that [Other P-415]children should be nurtured). This criterion aims to establish that a successful interpretation of minimal risk will expose children to a degree of nontherapeutic research risk that corresponds to commonly held ideas about the morally appropriate treatment of children, which, in turn, may facilitate the recruitment of parents willing to consent for their children’s research participation.

Third, a successful interpretation must meet the requirement of *defensibility*. It must constrain the degree of permissible risk to a non-arbitrary degree. It must offer persuasive reasons for why the risk threshold should be set at a particular level. Limiting the risks of nontherapeutic procedures to a defensible degree helps to ensure that clinical research will not sacrifice any particular child’s interests unduly for the sake of other children or for the social good. This requirement also helps to ensure that valuable research on children is not restricted unnecessarily. More generally, the requirement of defensibility helps to ensure that a successful threshold will be based on persuasive arguments. For an interpretation of minimal risk to be successful, these criteria must be met.

## 3. COMPETING INTERPRETATIONS

The criteria above help to establish the foundation for a successful interpretation of minimal risk. They also facilitate the examination of the following competing interpretations.

### The Risks of Routine Examination

One proposed interpretation suggests that minimal risk should be understood as the risks a healthy child faces during the performance of routine physical or psychological examinations or tests. According to this interpretation, minimal risk procedures would refer to the risks of a routine medical check-up, including routine age-appropriate physical and psychological examinations or tests, guidance and education, and immunizations (IOM 2004, 124). This interpretation is endorsed by Resnik (2005), Kopelman (2004, 366), and the Council for International Organizations of Medical Sciences’ *International Ethical Guidelines for Biomedical Research Involving Human Subjects* (CIOMS 2002, Guideline 9, 48).

One advantage of this interpretation is that it offers clear procedural guidance. The procedures involved in the routine examination of healthy children of different ages are reasonably well characterized (Kopelman 2004). Consequently, this interpretation facilitates the identification of the [Other P-416]kinds of risks that should be identified as minimal risk in research with children. But the routine examination interpretation does not meet the requirement of defensibility. The procedures administered during a healthy child’s medical examination are administered with the prospect of direct medical benefit. They track a child’s development and seek to identify any problems requiring follow-up or treatment (Wendler 2005). Given that the procedures of a routine examination involve risks undertaken with the prospect of direct medical benefit, they are not an obvious comparator for minimal risk, which constrains risks undertaken without corresponding benefit. There is no persuasive reason to think the risks of routine examinations of healthy children involve risks that would also be appropriate in research that does not aim to benefit a child. It follows that the risks of routine examination view is not a successful interpretation for the minimal risk threshold.

### Charitable Activities

David Wendler defends the “charitable activities” interpretation (2005; 2010; Wendler and Glantz 2007), which recognizes risks as minimal when they do not “exceed the risks of charitable activities deemed acceptable . . . in daily life” (2005, 40). That is, minimal risk refers to the kinds of risks that society would deem permissible for children to face while participating in charitable activities. For example, the risks of participating in Global Youth Service Day, in which children across the world carry out local community improvement projects including crop planting, visiting the sick, digging wells, and collecting donations (Wendler and Glantz 2007).

Wendler justifies this threshold by drawing parallels between research participation and participation in charitable activities. He points out that both charitable activities and nonbeneficial pediatric research are designed to help unrelated and unidentified others (Wendler and Glantz 2007). Neither activity is able to predict with certainty that it will benefit others; the benefits of a clinical trial, like those of a charitable fund raiser, may not materialize. But each is a valuable endeavor that offers a reasonable prospect of helping others (Wendler and Glantz 2007). Further, charitable activities—much like clinical research—are socially endorsed. Parents are encouraged to volunteer their children to help in the collection of charitable funds through carwashes or door-to-door collections (2005, 40). Given the similarities between charitable activities and clinical research, Wendler concludes that minimal risk should be understood as the risks of participating in charitable activities. [Other P-417]

Several aspects of this proposal are persuasive. The charitable participation standard meets the requirement of fidelity. It is consistent with the commonly held intuition that parents should encourage children to learn the value of helping others. Further, the charitable activities interpretation identifies a risk threshold based on activities that do not aim to offer direct benefit to the child, which improves on the routine examination interpretation.

However, the charitable participation interpretation faces limitations. It is overly restrictive, in two different ways. First, charitable activities are an overly narrow comparator activity for the risks of nontherapeutic procedures. Examples of charitable activities cited include the work of charitable organizations undertaken to help others in need, including the work of UNICEF and Habitat for Humanity (Wendler 2005; Wendler and Glantz 2007). These are instances of valuable activities undertaken or designed for the benefit of others. They are also activities in which children’s participation may be encouraged; charity work helps to teach children socially and morally valuable character traits. But charitable activities are only one instance of the kind of valuable activities in which society is willing to expose children to risk without direct corresponding benefit.

Consider other activities, such as promoting religious harmony. One might contribute to the promotion of religious harmony by organizing local activities in which children are encouraged to learn about the history and customs of different religions, to attend religious ceremonies, and to participate in religious holidays. These activities contribute to the desirable goals of promoting harmony and peace, both in communities in which intolerance is prevalent and in the wide implementation of these programs as educational or prevention measures. These are valuable activities in which to encourage children’s participation for the benefit of us all.^3^ But activities designed to promote religious harmony are not well characterized as instances of charity work. This is the case for a variety of valuable activities, including activities undertaken to promote public health, to promote democracy, to reduce pollution, or to promote racial and religious harmony. The charitable activities interpretation aims to capture the risks, without corresponding benefit, that children should be permitted to undertake for the sake of others, but it identifies only a subsection of the activities that seem permissible. If follows that this interpretation may unduly constrain the kinds of risks that should be permitted in research. Ultimately, a successful minimal risk threshold must be robust enough to accommodate a broader range of activities. [Other P-418]

Further, the charitable participation standard encounters a second pressing problem. It does not meet the requirement of generality. Infants and very young children lack the capacities required to undertake and to understand altruistic behavior. These children do not (and are generally not expected to) participate in charitable activities. This idea is reflected in the rules of charitable organizations, which often restrict the kinds of activities that may be undertaken by children of different ages (Wendler and Glantz 2007). For example, Habitat for Humanity permits only children 16 years old or older on construction sites. They also outline age-appropriate activities for children between 5–16, but they exclude all children below the age of five. It follows that endorsing this interpretation of minimal risk would exclude infants and very young children from research.

This problem is noted as a disadvantage of the interpretation (Wendler and Glantz 2007, 580), but it is a serious problem. Applying this interpretation seems to prohibit the progress of research that investigates the medical treatment of infants and young children, which would likely compromise their medical care. Further, it is not clear how this interpretation would permit current research on infants and very young children.^4^

### Risks in Family Life

Terrence Ackerman defends a different interpretation according to which a “research procedure involving minimal risk is one in which the probability of physical and psychological harm is no more than that to which it is appropriate to intentionally expose a child for educational purposes in family life situations” (1980, 106). That is, the degree of risk permissible in nontherapeutic research procedures should be restricted to the degree of risk to which parents are willing to intentionally expose their children for the purposes of their moral education in family life.

Ackerman develops this interpretation by pointing out that parents may, at times, legitimately intervene into the lives of their children. And these interventions may be permissible even when they do not aim to benefit the children themselves. There are several instances in which interventions into a child’s life are permissible in the absence of corresponding benefit. It is permissible for parents to intervene in the lives of their children to inculcate traits of character that contribute to a child’s development into a morally acceptable adult (1980, 95–96). That is, parents may intervene to teach a child shared moral duties, such as showing respect for others, keeping promises, and avoiding harm. Further, it is permissible for parents to [Other P-419]intervene in the lives of their children when doing so promotes the interests of others, especially family members. For example, it may be reasonable for parents to move a child away from established social relationships to take up a new job in a different country or to force a child to wait long hours in an emergency room while her sibling’s broken bone is attended to (1980, 96). That is, it is permissible for parents to expose children to some risk without benefit, when doing so promotes the interests of other family members or the interests of the family as a whole.

Ackerman extends this analysis of family life to nontherapeutic research procedures with children. He argues that if certain kinds of interventions that are not in a child’s direct interests are morally legitimate in family life then they can, by analogy, also be morally justified in the context of research with children. Given that clinical research—much in the same way as children’s moral education—contributes to a socially worthy goal, it seems reasonable to allow the risks permitted in children’s moral education in family life in nontherapeutic research procedures (1980, 106). Interventions in the family setting that promote a child’s moral education include chores such as washing the dinner dishes, which involves some risk of cuts and the discomfort of not doing other things during that time, and participating in an outing that is not of immediate interest to a child, such as a trip to the grocery store (1980, 107).

Ackerman’s proposal persuasively identifies an area in which it is permissible to expose children to some risks without benefits to the child. One advantage of Ackerman’s interpretation is that it has a broader scope than the charitable activities interpretation of minimal risk. The risks to which it is permissible to expose children for educational purposes in family life include not only charitable activities, but also risks undertaken in the interests of the family or for other socially valuable causes.

However, there are several limitations to drawing the upper threshold as the risks to which parents intentionally expose children for educational purposes in family life. One might understand this proposal as drawing the upper limit as any risks permitted by parents in family life that fall below the legal threshold allowed by abuse and neglect laws. Understood in this way, the threshold seems overly permissive. There is value to permitting a wide degree of parental discretion before requiring state intervention in family life (Ross 1998). But replicating this threshold in the context of clinical research is less desirable (Wendler 2010, 90). These laws call for intervention only in order to prohibit parents from treating children in particularly undesirable ways. In order to encourage research enrollment [Other P-420]and to contribute to public trust in research, it is not sufficient to draw a threshold that permits any type of intervention that would not warrant state intrusion in family life (including interventions that fall just short of abuse and neglect laws) (Wendler 2010). Instead, it is preferable that there should be a separable or independent reason to think that the threshold permits only interventions that should be acceptable.

Ackerman’s proposal is perhaps better understood as indexing permissible risks to the experiences to which parents *should* expose children for their education in family life (rather than to those interventions that are legally tolerated). On this understanding, the threshold likely falls well below the abuse and neglect threshold. It reflects a social judgment about the kinds of risks that are permissible in family life situations (Nelson and Ross 2005, 565).

However, restricting the interpretation to the risks to which parents should expose children does not offer sufficient guidance about which risks of family life should be permitted. The problem is that there exists no unambiguous body of risks to which it is socially permissible to expose children for educational purposes in family life. There exists a broad spectrum of different parenting styles and no common social judgment about the kinds of risks that are permissible for children’s moral education in the family setting. Further, good parents are likely to disagree about what is appropriate for children. Some emphasize the importance of programs of strict education, while others endorse the importance of play time. And it is not clear how to mediate between these parental judgments about the appropriate treatment of children.

One instance of this sort of difficulty can be found in the life of John Stuart Mill. James Mill required John Stuart Mill to learn Greek at the age of three, Latin at age eight, and to study the basics of economic theory complemented by extensive work in logic and mathematics by age fourteen. Acquiring this knowledge is admirable, but Mill complained that his father’s rigorous training led to the intense depression he suffered by age twenty (Wilson 2007). The problem is that it is not clear how to deal with this kind of example. There is little question that James Mill’s methods were severe. But they also contributed to John Stuart Mill becoming one of the most influential English-speaking philosophers of his time (Wilson 2007).

More generally, it is not clear where to draw the line within the spectrum of risks to which parents intentionally expose their children for the sake of their moral education in the family life setting. Given the divergence between the ways in which parents educate their children in [Other P-421]family life, it is not clear whether the threshold for acceptable risk should be the degree of risk permitted in a child’s moral education by very strict educators or the practices endorsed by parents who place little value on a young child’s education. Different perspectives may contribute to widespread variation between determinations about the permissibility of nontherapeutic procedures and could contribute to problems in assessing the permissibility of risks in multi-site studies. Further, it is not clear how to resolve conflicting judgments about the risks that are permissible for a child’s education in family life.

Another limitation with Ackerman’s interpretation is that it does not meet the requirement of generality. Parents do not generally morally educate infants or very young children. It follows that the risks in family life interpretation offers no persuasive reason why it is permissible to include infants and very young children in research that exposes them to risks for the benefit of others. Overall, this interpretation has considerable merit, but its success depends on a clearer account of what should count as appropriate parenting and of what should be permitted for the sake of children’s education in family life. It also requires an explanation of the ways in which we morally educate very young children.

## 4. THE RISKS-OF-DAILY-LIFE INTERPRETATION

The fourth interpretation, and the one I wish to defend, proposes that minimal risk should be understood as the risks of everyday experiences (henceforth the risks-of-daily-life).^5^ This interpretation is based on the idea that no matter how careful a person is, carrying out her usual daily activities—such as eating a meal or crossing the street—involves exposure to some risk. And this inevitable degree of risk should be understood as minimal risk (Ackerman 2001, 32).

There is little question that our daily activities expose us to risks, but what, if anything, is morally relevant about the risks encountered in daily life? Specifically, why should the risks faced daily inform the permissibility of the risks of nontherapeutic procedures? A successful explanation for why children’s daily risks are an appropriate measure for the permissibility of nontherapeutic research procedures involves two main tasks: The justification must explain which group of children’s daily experiences should be captured in a risks-of-daily-life interpretation. And it must also explain why the daily experiences of a particular group of children should be the appropriate comparator class of activities for risks without benefit in clinical research. [Other P-422]

These two aspects of a defense of the risks-of-daily-life interpretation are perhaps best understood as addressing two related questions: First, whose daily life should be captured by the minimal risk threshold? Second, what, if anything, is morally relevant about the daily risks of a particular group of children? That is, why are daily risks an appropriate comparator activity for the risks of nontherapeutic research procedures? I will offer a brief summary of my answer to the first question, and then focus on the second question, which has not received adequate attention.

The first question—whose daily life should be captured by the minimal risk threshold?—is motivated by the idea that in the absence of a specified reference group, it makes little sense to discuss the risks of daily life. The threshold might refer to the risks faced daily by healthy children, sick children, average children, children living in dangerous settings, or some combination of different groups of children. To put it another way, one problem with the risks-of-daily-life interpretation is that it is ambiguous (Kopelman 2000). This ambiguity is problematic because the daily risks of different groups of children are likely to vary (sometimes considerably) and a minimal risk threshold that does not constrain variable treatment is liable to permit unfair treatment for some children in research (Kopelman 2004; Evans and Evans 1996, 66). For example, if the risks-of-daily-life interpretation could be used to refer to the high daily risk of severe injury or death faced by an unfortunate group of children—such as children living in a war zone—then it might be used to justify comparably high risks in research without benefit, which would be unjust (Kopelman 2004). To resolve this problem and to avoid injustices, one must explain which group of children’s daily lives should be captured by the minimal risk threshold.^6^

Commentators typically offer one of two answers to the “whose daily life?” question. They argue that minimal risk should refer to either the daily lives of healthy children^7^ or that minimal risk should refer to the daily lives of the subjects of the research, which includes all children eligible for study participation (including both healthy and sick children). Elsewhere, I have argued that neither of these answers is persuasive and that the risks-of-daily-life should refer to the daily risks of children who are *not unduly burdened*, which is understood as referring to children who fare well (Binik 2014). According to this argument, children fare well when they possess high degrees of the substantive goods of health, biological needs, intellectually engaging activities, meaningful relationships, play time, bodily integrity, and happiness (Binik 2014). When discussing the risks-of-daily-life, I use it to refer only to the daily experiences of children [Other P-423]who are not unduly burdened—that is, children who possess high degrees of these goods. By constraining the discussion of the risks-of-daily-life threshold to the daily experiences of children who are not unduly burdened, I aim to help resolve the problem of ambiguity. To put it another way, constraining the daily life interpretation to a particular group of children helps to answer one question raised by the risks-of-daily-life interpretation.

However, the current goal is not to defend this view of children’s welfare.^8^ The point I wish to emphasize is that addressing the problem of ambiguity by explaining whose daily life should be captured by the minimal risk threshold is insufficient to justify a risks-of-daily-life threshold. That is, specifying whose daily life is captured by the minimal risk threshold is not—on its own—a sufficient justification for the risks-of-daily-life interpretation. Even if one accepts the idea that the risk threshold refers to the daily lives of children who are not unduly burdened, more needs to be said about why the daily risks of children who fare well serve as a useful comparator for the moral threshold for acceptable risks in nontherapeutic research procedures. That is, an explanation needs to be offered about what, if anything, is morally relevant about the everyday risks of a particular group of children (whether it refers to flourishing children, healthy children, or any other group of children). In what follows, I focus on this second aspect of the defense of the risks-of-daily-life interpretation, which has received less attention. I argue that the justification currently offered is inadequate and I propose a new defense of the risks-of-daily-life threshold.

### Current Justification

What, if anything, make the daily experiences of some particular group of children a justifiable comparator for risks in research? One prominent answer to this question merits consideration. To justify the risks-of-daily-life threshold, some have appealed to the notion of risk replacement (Freedman, Fuks, and Weijer 1993). The idea is that if we limit the risks of nontherapeutic procedures according to the risks a child faces in her usual activities, then the child’s participation in a trial will not increase the amount of risk she undertakes at any given time. The child will undertake the risks of research, but she will forgo the risks of activities she would otherwise be pursuing. To put it another way, the research risks substitute, but do not add to, the risks a child would otherwise face at a particular time (Freedman, Fuks, and Weijer 1993; Hope and McMillan 2004; Ackerman 2001). The rationale is that restricting the [Other P-424]risks of nontherapeutic procedures to the risks-of-daily-life is appropriate because it protects children from facing additional harms as the result of their research participation.

While persuasive in some instances, the risk replacement argument cannot be applied to the justification of all nontherapeutic procedures on children. The problem is that it argues from the idea that a child cannot simultaneously undergo a nontherapeutic procedure in the research setting while she is also engaging in another activity to the conclusion that the child will not experience the risks of both experiences simultaneously. That is, the argument draws on the idea that a child cannot undergo a nontherapeutic blood draw at the same time that she is playing in a playground to suggest that the risks of the blood draw replace the risks of playing outdoors. But this does not always follow. It is true that a child cannot physically be in multiple places at one time, but she may well experience the risks of different kinds of activities simultaneously. That is, while one activity may replace the other, the risks of nontherapeutic procedures will not always substitute the risks of other daily experiences.^9^

Risks are not always substitutive in this sense because the risks associated with some procedures are not constrained to the period of time during which the procedure is administered; they may persist after a trial. For example, a child may continue to experience the effects of an investigational drug after she leaves the clinic and goes cycling. In this instance, the child would face the risks of the investigational drug and the risks of cycling simultaneously. And if the risks of the activities are not always interchangeable, then research participation may increase (rather than replace) a child’s usual risks. More generally, in some cases, the activities undertaken by a child post-procedure but while the effects of an intervention persist do not replace but add to a child’s daily risks.

Proponents of the risk replacement justification would likely agree with this specification. The original text argues that the research risks are “*to a degree* substitutive, rather than additive” (Freedman, Fuks, and Weijer 1993, 17, emphasis added), suggesting that the argument was not intended to apply in all instances. Perhaps the most plausible reading is that the risks-of-daily-life threshold may, at times, be justified because it helps to ensure that research risks replace without adding to a child’s experiences, but that this argument alone is inadequate to justify the broad use of a risks-of-daily-life threshold in pediatric research.

Some commentators also suggest a second defense of the risks-of-daily-life threshold. They claim that this threshold reflects “a level of risk that [Other P-425]is not simply accepted but is deemed socially acceptable,” which is to say that daily risks may be an appropriate comparator because they have normative force (Freedman, Fuks, and Weijer 1993, 17). They reflect the set of experiences that are judged to be acceptable for children.

One might object to this defense by pointing out that over the course of a day, many children face risks—such as extreme and persistent bullying or abuse—that are not thought to be acceptable, but that simply haven’t been successfully eliminated from some children’s lives (Wendler 2010, 69). This objection suggests that daily risks are not morally relevant risks for children. This problem can be mitigated by combining the defense of the risks-of-daily-life concept with a specification of whose daily life should be captured by this threshold. Combined with the *undue burden* argument, the justification is the following: Restricting nontherapeutic research risks according to the daily experiences of children who flourish is justifiable because flourishing children live good lives, that is, their lives involve acceptable (or at least permissible) experiences. By restricting the threshold to children who are not unduly burdened rather than to another standard, such as healthy or normal children, the idea that daily risks have normative force becomes more compelling.

But appealing to the normative significance of daily experiences to justify the risks-of-daily-life threshold raises other problems that have not been addressed. For instance, it is not clear that we actively and accurately consider the risks of our common experiences. Most undertake daily experiences without much calculation, which suggests that these activities may be accepted, but does not indicate that they are considered acceptable. Moreover, it is not clear why these risks should be understood as normatively significant. That is, it is not clear what grounds the normativity of daily risks.

Where does this leave us? I’ve argued that three of the most prominent interpretations of minimal risk are inadequate. The fourth interpretation, the risks-of-daily-life interpretation, is promising but it cannot rely on a specification of whose daily life it captures alone. It must also explain what, if anything, is morally relevant about the risks of daily life. The risk replacement argument is a persuasive justification when research risks are constrained to the duration of the study procedure, but it does not apply in all instances. In instances when this argument does not apply, the idea that daily risks have normative force carries intuitive appeal, but more needs to be said about why daily risks are acceptable, rather than simply ignored or misunderstood. Accordingly, the success of the risks-of-daily-life [Other P-426]interpretation depends on a new defense that explains why daily experiences are normatively significant.

### A New Defense of the Moral Relevance of the Risks-of-Daily-Life

I will defend the idea that daily risks are a justifiable comparator activity for the minimal risk threshold. I will argue that these risks are morally relevant; the risks of daily life are accepted and should be accepted because they are part of a reasonable trade-off between personal safety and our ability to pursue meaningful lives. That is, they reflect the kind of sacrifice that is reasonable to undertake in the interests of pursuing the kinds of lives we want to live as a community. I will then argue that insofar as daily risks reflect the sorts of risks we wish to take in the interests of broader social values, they offer a justifiable interpretation for minimal risk.

The idea that daily risks are accepted by most is well reflected in our behavior. Most people—irrespective of whether they are risk-averse or risk-takers—undertake the risks of daily activities without much thought or calculation. Further, we identify those who disagree with this judgment, that is, people who try and avoid daily risks, as different. For example, we understand agoraphobes—who experience anxiety about being in situations or places that make escape difficult and avoid daily activities such as riding in an elevator, travelling in a bus, riding in a car, or walking through crowded public places—as suffering from a disorder (DSM-V 2013). We classify their behavior as unusual and recommend therapies to help them face everyday experiences without difficulty.

This suggests that daily risks—including crossing the street, riding in public transportation, eating a meal, and entering public areas—are generally thought to be safe enough. I take this to be reasonably uncontroversial; most people seem to undertake the activities of daily life and their corresponding risks without much thought, concern, or calculation. This suggests that daily risks are socially accepted. But the more difficult question, and the one on which I will focus, is should we behave as we do? That is, do we have good reasons to accept daily risks? Is there something socially acceptable or morally relevant about our daily activities?

One possibility is that we do not have good reasons to accept the risks of our everyday experiences. Our acceptance of daily activities is simply the result of a failure to properly understand the risks posed by our usual activities. A substantial body of evidence from cognitive psychology suggests that we are notoriously poor at judging risks (Tversky and Kahneman 1981; 1974[Other P-427]; Slovic, Fischhoff, and Lichtenstein 1982; Teuber 1990). We assess risks by appealing to a series of common heuristics that lead to systematic errors of judgment.

This evidence suggests that we tend to judge the likelihood of an event based on whether it is available to us, that is, based on whether instances of this event can be easily recalled or imagined. For example, people often guess the likelihood of heart attacks in middle-aged men based on their recollection of these occurrences amongst acquaintances (Tversky and Kahnmenan 1982; Slovic, Fischhoff, and Lichtenstein 1982). But this judgment is problematic; it ignores the fact that proximate or recent instances of events are not always good indicators of the most pressing dangers (Slovic, Fischhoff, and Lichtenstein 1982). Another common error arises in our estimation of probabilities. We tend to begin with an initial value and then adjust this value to arrive at a final answer. But these adjustments are insufficient; different starting points—that are generally the result of a particular formulation of a problem or the result of a partial computation—yield different estimates that are biased toward the initial values. Consequently, our estimations of probabilities often contribute to distorted risk perceptions (Tversky and Kahneman 1982).

Further, we overestimate rare causes of death and underestimate common causes of death (Teuber 1990; Slovic, Fischhoff, and Lichtenstein 1982), we undervalue outcomes that are probable in comparison with outcomes that are obtained with certainty (Kahneman and Tversky 1982; Tversky and Kahneman 1981), we are biased to think bad things will happen to others but not to us (Slovic, Fischhoff, and Lichtenstein 1982), and we fall prey to the gambler’s fallacy, that is, the belief that chance is a self-correcting process in which a deviation in one direction leads to a deviation in the opposite direction in order to maintain an equilibrium (Teuber 1990; Tversky and Kahneman 1982). Further, research shows that even people with extensive training in statistics are prone to intuitive biases of risk perception (Kahneman and Tversky 1982).

The evidence from social psychology is extensive and persuasive; we are notoriously poor at calculating the risks associated with our usual activities. And this raises an important question: what are the implications of this evidence for the plausibility of a risks-of-daily-life threshold in pediatric research? That is, what are the implications of this evidence for the suggestion that the risks-of-daily-life are morally relevant? One might understand this evidence as suggesting that we do not have good reasons to think that daily activities involve acceptable degrees of risk. According to [Other P-428]this interpretation, our acceptance of the risks of daily activities is the result of mistakes in our risk judgements or perhaps a psychological mechanism designed to deal with the inevitability of encountering the risks of our common experiences. Further, this interpretation suggests that if we had a better understanding of the dangers posed by our everyday activities, we would not readily endorse them in the absence of personal gain.

Wendler argues that our acceptance of (or at least the fact that we ignore) the risks of daily life is not a fully rational process (2010, 66). He argues that “[r]ecognizing the different factors that influence our perception of risks undercuts the attempt to ascribe moral significance to the threshold for which risks we attend to” (2010, 67). He emphasizes that “although our attitude toward the risks of everyday life makes sense psychologically, it does not provide an ethical justification for exposing children to the same level of risks” (2010, 66). That is, daily risks should not be understood as a morally relevant comparator for risks in research with children. Further, he suggests that more empirical data about the risks of daily experiences is needed to facilitate determinations about the permissibility of risks (Wendler et al. 2005).

I wish to propose an alternative interpretation. It is clear that we assess risks in ways that are predictably problematic and lead to systematic errors in the calculation of the degree of risk involved in our everyday experiences. But understanding risk perception studies as demonstrating the irrationality of people’s attitudes towards risk may reflect an overly narrow understanding of rationality (Teuber 1990, 248). That is, these studies should not be understood as suggesting that we are mistaken in our endorsement of the acceptability of everyday risks. The ways in which we characterize the risk of everyday experiences are perhaps better understood as suggesting that we are not uniquely motivated by the degree of risk involved in an activity. That is, we do not seek to limit our activities to those that pose very low degrees of risk and to exclude all higher-risk activities. Nor do we seek consistency in the degrees of risk that should be deemed permissible.

Instead, we manipulate our perception of risk to line up with the kinds of activities we think are valuable. That is, we accept the permissibility of the risks of certain activities because they reflect activities that are socially valuable. As a society, we value certain projects, goals, or ways of life. These projects, goals, or ways of life are valued for the benefit of society as a whole and not uniquely for a particular individual. Our decisions to focus on some risks and to disregard others should be understood—not as [Other P-429]irrational—but as a reflection of the ways of life cherished by the group, that is, as a reflection of the activities that are socially endorsed.

The idea that our risk perceptions are, at least in part, a reflection of societal values helps to explain why we have good reasons to accept the risks of daily life. We deem these risks acceptable because they help to uphold valuable ways of life. Understood in this way, our acceptance of daily risks as permissible reflects the group’s conception of the kinds of projects and institutions that help to uphold and promote meaningful ways of life. To put it another way, the risks of daily activities are a reasonable sacrifice to undertake in the interests of pursuing the kinds of lives we want to live as a society.

This interpretation corresponds to the ways in which we make decisions about the permissibility of actions and about the permissibility of children’s participation in different kinds of activities. For instance, when considering whether a child should be taken on a car trip to the grocery store, most parents do not look up the statistics of traffic accidents or become fixated on the likelihood of a disaster. Instead, the sorts of considerations taken into account are whether the trip is an efficient usage of time or whether groceries are required. Further, even if the statistics of road accidents were researched, learning that we had significantly underestimated the risks of car travel for children seems unlikely to motivate a change in the behavior of most families.

This suggests that our acceptance of the risks of daily life may not be the result of our propensity to misjudge risks and deem risky situations safer than they really are. Instead we aim to consider whether an activity (and its accompanying risks) contributes to or upholds useful ways of life. More generally, it seems unlikely that learning empirical statistics about the likelihood and magnitude of the harms of daily life would convince us that our usual activities are too risky. We accept daily risks because we consider them a reasonable trade-off between safety and valuable ways of life.

This interpretation is consistent with the idea that we often make mistakes in our risk calculations. And it offers a more optimistic view of our decision-making procedures. Rather than understanding the acceptance of everyday risks as the result of our propensity to make ill-informed judgments or as reflecting a mechanism for coping with our inability to avoid common risks, it suggests that there is something valuable about everyday experiences—at least those in flourishing societies—and that our decisions to focus on some risks and to disregard others reflect the ways of life cherished by a community. [Other P-430]

### Objection and Response

One might object to this argument by claiming that it mischaracterizes the reasons for which we accept the risks of daily life. The risks of everyday experiences are not considered acceptable because they are morally relevant, but because these experiences offer corresponding benefit. That is, they are a reasonable trade-off between risks and direct benefits to an individual (Wendler 2010, 68). For example, the risks of playing in a playground may be considered permissible because they are offset by the benefits of outdoor exercise or playing with friends. This objection is pressing because the minimal risk threshold seeks to identify a set of risks that are permissible to undertake in the absence of corresponding benefit. If daily activities are acceptable only because they offer benefit to an individual, then they would be an inappropriate comparator for risks without benefit in research with children.

This is an important objection, to which I offer two responses. First, it is certainly true that some of our daily activities offer corresponding benefit to the individual. But this is not necessarily problematic. My view is consistent with the idea that some activities of daily life offer corresponding benefit, provided that we recognize that our daily activities are many, varied, and complex. Some activities offer corresponding benefit (such as learning to ski) while other activities do not (such as taking a child along for the drive on a work-related errand). This suggests that daily risks are undertaken and accepted for a set of complicated reasons, many of which do not involve benefit for an individual child and that the minimal risk threshold is perhaps best understood as everyday experiences that are acceptable even when they do not offer the prospect of direct benefit.

Second, the acceptance of daily risks as a meaningful trade-off between personal safety and valuable ways of life occurs not only at the individual level, but also at the group level. And while trade-offs at the individual level may offer corresponding benefit, this is less often the case at the group level.^10^ Specifically, certain activities are considered valuable at the group level. There are group conceptions of the kinds of projects and institutions that help to promote valuable ways of life. For instance, environmental goals, such as reducing pollution, are often recognized at the group level. It is for this reason that many societies sanction certain behaviors (e.g., charges for plastic grocery bags) and encourage activities such as community clean-up days and household recycling.

Pursuing environmental goals does not aim uniquely to offer direct benefit to the individual. It aims to improve the world for future people. [Other P-431]Activities undertaken to improve the environment involve some amount of risk; recycling exposes us to risks of cuts while sorting materials and the risks of moving recyclables to the curb. And evidence suggests that if we were to estimate the risks involved, we would likely fall short of the mark. But when considering whether we should participate in household recycling and whether we should encourage children to recycle, most do not calculate risks quantitatively.

Part of the explanation for this is that we accept the risks of activities undertaken in the interests of commonly valued social goals. We accept and uphold these risks because they are consistent with values we wish our communities to uphold. That is, they are a reasonable trade-off to make in order to pursue the kinds of lives we want to live. These activities are not found acceptable uniquely because they offer direct benefit (though some may), but because they contribute to meaningful ways of life.

### Clarifications

Several additional points bear clarification. The first concerns the role of empirical evidence in the justification for the risks-of-daily-life interpretation. The idea that our risk perceptions are linked with and formed by our values is not a rejection of empirical information about risk. The argument is not that empirical evidence never motivates changes in our behavior. Statistics about the risks of death or injury involved in common activities are useful in drawing attention to under-recognized or unanticipated risks of common activities. And they should be recognized by a successful interpretation of minimal risk.

My view is consistent with the idea that empirical evidence about the risks of common activities may be strong enough to persuade people that a given activity should be reconsidered. In these instances, the activity would no longer be considered a reasonable trade-off between personal safety and valuable social goals and should not be accepted in the absence of benefit. The claim I do wish to emphasize is that restricting permissible risks according to a quantitative measure is, at least to some extent, arbitrary. Learning that recycling poses a 1/1000 risk of a cut offers no straightforward reason about whether a child should be encouraged to recycle. Part of the explanation for this is that our decisions about the permissibility of actions are complex; these decisions reflect our beliefs about what makes life valuable. Understood in this way, my account of minimal risk as the risks-of-daily-life may be informed, but not primarily guided, by empirical evidence about the risks of our common activities. [Other P-432]

A second point worth clarifying addresses the meaning of the “risks-of-daily-life.” Many do not undertake socially valuable causes, such as participating in a peace rally, on a daily basis. I criticized the charitable activity interpretation of minimal risk for its inability to accommodate this sort of activity, but one might question whether the risks of participating in an occasional rally for a valuable cause fit more comfortably on the risks-of-daily-life proposal. This tension can be resolved by clarifying the meaning of “risks-of-daily-life.” This standard should not be understood as referring to a body of risks that are literally faced every day by all flourishing children. There is likely no such body of risks. Instead, it refers to a background range of risks that a child who is not unduly burdened may encounter on any given day (Miller and Weijer 2005, 39). This aims to include commonly encountered risks, such as the risks of crossing the street, as well as the risks of occasional daily experiences, such as participating in a peaceful rally under parental supervision.

A third valuable question to consider is whether the risk threshold should be constrained to a subset of daily risks—the risks of daily activities that we make little attempt to minimize. This question is motivated by the recognition that the risks of some of our common experiences are accepted with little attempt to avoid them, while we go to some trouble to minimize the potential harms of other daily activities. For example, when children are asked to recycle as part of a socially valuable activity, they are not routinely asked to wear gloves, but when riding in a car on the way to a community clean-up day, they are required to wear seatbelts or ride in car seats. Given the efforts made to limit the risks of some daily activities but not others, it is worth considering whether, and if so how, this should be accounted for in a risks-of-daily-life threshold.

One might understand our behavior as suggesting that the risk threshold should be restricted to only daily experiences that are acceptable as they are (e.g., recycling but not car travel). But another plausible way of accommodating differences in our treatment of everyday activities is to understand the threshold as referring to daily activities undertaken in the way that is deemed acceptable (e.g., car travel with a seat belt). This understanding would track people’s reasonable attempts to minimize the risks of daily activities while also accounting for the idea that once we’ve restricted the risks of some activities (even those with the potential for severe consequences), they are often considered acceptable in the interests of socially valuable causes. [Other P-433]

Similarly, the administration of some nontherapeutic research procedures involves deliberate efforts to minimize their risks, for example, sterilizing a needle before a blood draw or requiring adequate training for researchers before they interview children about potentially sensitive issues. But this need not suggest that these procedures are impermissible. Instead, the risk threshold should track the ways in which we treat the risks of our daily experiences without eliminating them from the set of comparator activities.

A final point to clarify is that ascribing moral significance to the risks-of-daily-life does not suggest that the daily experiences of every child are acceptable. This justification does not endorse the acceptability of the daily experiences of children who are exposed to high risks as the result of circumstances outside of their control (e.g., serious disease, dangerous living circumstances). The analysis is restricted to the daily lives of children who are not unduly burdened, understood as children who fare well (Binik 2014). In other words, this defense operates in combination with an account of whose daily life should be captured by minimal risk. Only the daily risks of children who fare well should be used as the comparator for risks without benefit in research. Examining whose daily life should be captured by the risk threshold and examining what, if anything, is morally relevant about the daily experiences of a particular group of children are complementary aspects of an interpretation of minimal risk.

What are the implications of this argument for research with children? Ascribing moral significance to the risks-of-daily-life informs the threshold for permissible risks in pediatric research. Clinical research is an instance of a valuable social cause in which participants are often required to undertake risks without corresponding benefit. I have argued that the risks of daily life reflect the risks that should be found acceptable in the pursuit of valuable social causes. If successful, this argument suggests that daily risks should serve as an appropriate comparator for risks without benefit in pediatric research. That is, minimal risk should be interpreted as the risks-of-daily-life. Research risks without benefits should be constrained to the risks of daily life because this threshold promotes a reasonable tradeoff between personal safety and our ability to pursue meaningful lives.

## 5. COMPETING INTERPRETATIONS RECONSIDERED

How does the proposed view differ from and resolve problems with the competing interpretations of minimal risk? The proposed defense of the risks-of-daily-life interpretation improves upon the risks of routine examination interpretation by appealing to a comparator activity [Other P-434]that is not uniquely undertaken because of its ability to provide direct corresponding benefit to a child.

The second view considered was the charitable participation interpretation. There are commonalities between this view and the above proposal. Each endorses a comparator activity that is also valuable in the absence of corresponding benefit to a child. But the risks-of-daily-life interpretation does not refer uniquely to activities designed or undertaken for the benefit of others. It also includes activities that offer broad social value that benefits us all. As a result, the risks-of-daily-life interpretation also accommodates children’s occasional participation in a wider range of socially valuable activities than the charitable activities standard, including the promotion of public health and the promotion of religious harmony. The risks-of-daily-life view also offers an explanation of why common mistakes in risk judgments do not necessarily suggest the impermissibility of comparing research risks without benefits to risks in daily life.

The third interpretation considered was the risks undertaken for a child’s moral education in family life view. Much like this view, the risks-of-daily-life account endorses the central role of parents in determining what should be permissible for children, but it does not rely uniquely on ambiguous social judgments about what should count as permissible over the course of a child’s moral education in family life.

Further, both the risks in family life view and the charitable participation interpretation exclude infants and very young children from research. The proposed defense of the risks-of-daily-life threshold helps to resolve this problem by endorsing a comparator activity that recognizes a role for infants and young children as research subjects.

More generally, the risks-of-daily-life threshold endorses a different normative foundation for the permissibility of risks in research without benefit. It does not derive normative force from the transparency of the procedure of a medical check-up or from the similar structures of activities undertaken in charity work and research risks undertaken for the sake of others. Nor does it rely exclusively on a common social judgment about acceptable parenting. Instead, it derives its normative force from the idea that there is something valuable about life (and its accompanying risks) in societies in which children flourish. This proposal offers a justification for why daily risks may be thought of as morally meaningful (rather than unavoidable or the result of mistakes in our risk judgment).

In spite of these differences between various interpretations of minimal risk, it is worth emphasizing that it is an advantage of this defense of the [Other P-435]risks-of-daily-life interpretation is that it is consistent with alternative interpretations. This view aims to specify and to build on the merits of differing interpretations while helping to resolve their shortcomings. For instance, this defense of the risks-of-daily-life threshold is continuous with the risks of family life view. After all, children’s daily experiences are (to some extent) those experiences permitted by their parents in family life. The proposed interpretation is also consistent with the charitable activities and the risks of routine examination interpretations. It recognizes each of these as examples of the daily activities of flourishing children. That is, this interpretation endorses many of the activities that fall under each of the alternative interpretations as examples of the sorts of daily risks that should also be found permissible in nontherapeutic research procedures. But it is better able to capture the set of experiences that are permissible for children to undertake in the absence of direct corresponding benefit.^11^

### The Necessary Criteria

This defense of the risks-of-daily-life interpretation is well equipped to meet the necessary criteria for a successful interpretation. First, the risks-of-daily-life interpretation meets the requirement of *generality*. Children of all ages experience the risks of daily life. The daily activities of an infant and a nonautonomous teenager differ considerably, but each group undertakes an age-appropriate set of experiences. For instance, infants may face risks of minor bruises or bumps while being pushed in a stroller or some temporary discomfort generated by environmental factors, including loud noises. Young children face a different set of daily experiences (and accompanying risks), including those generated by outdoor games with other children. By identifying daily experiences as the comparator activity for research risks, this account endorses the inclusion of children of all ages in research. And it does so in a way that is sensitive to differences between different age groups of children. Accordingly, this interpretation satisfies the generality requirement.

Interpreting minimal risk as the risks-of-daily-life also meets the requirement of *fidelity*. Defending the moral relevance of the risks-of-daily-life lines up with the commonly held belief that the daily activities of flourishing children are permissible (even when we lack empirical data about the amount of risk each of these activities involve). It also reflects the widely held belief that parents should have wide latitude in determining what sorts of activities their children should undertake on a daily basis. [Other P-436]

I’ve suggested two reasons why this interpretation meets the third requirement—*defensibility*. In some instances, research risks replace the risks of activities that would otherwise be undertaken. In these instances, the risks-of-daily-life threshold is defensible because it helps to ensure that research participation will not increase the amount of risk a child will face at some particular time (Freedman, Fuks, and Weijer 1993). But I’ve also argued that there exist instances when the risks of daily activities and the risks of research procedures are not interchangeable. In these instances a different defense is necessary. I have proposed that in these instances, the daily risks of flourishing children are defensible because they are a reasonable trade-off between personal safety and our ability to pursue valuable lives.

## CONCLUSION

Finally, I have argued that the ethical justification for children’s inclusion in research depends on identifying a defensible threshold for research risk without corresponding medical benefit. This threshold may be captured by the minimal risk threshold—a central feature of national and international guidelines concerning the ethical conduct of research and competing systems for the ethical assessment of research risks. I argued that the risks-of-daily-life interpretation of minimal risk has potential, but should not be defended uniquely according to a risk-replacement argument. Rather, daily risks should be understood as morally relevant because they offer a reasonable trade-off between personal safety and valuable life. This interpretation retains the merits of competing views, but is better able to meet the necessary criteria for a successful understanding of minimal risk.

## NOTES

1
The minimal risk threshold plays a different role in differing systems for risk assessment. At times, it is understood as constraining research that does not offer the prospect of benefit. For example, see Rid and Wendler (2011), and Wendler (2006). Others understand minimal risk as governing the permissibility of nontherapeutic research procedures, which are undertaken in all clinical trials involving children (including those that offer some prospect for direct medical benefit). For instance, see CIOMS (2002), NBAC (2001), Weijer and Miller (2004), and Weijer (2000).

In this paper, I use minimal risk as a constraint on the risks of nontherapeutic procedures. But it is worth emphasizing that irrespective of differences between competing systems of risk assessment, each endorses the use of an upper limit to risk without benefit in research with children. It follows that my proposal for the minimal risk threshold is relevant for multiple systems for risk assessment.

2
In keeping with much of the regulatory language and the bioethics literature, I refer to the upper threshold for permissible risk without benefit in research with children as the minimal risk threshold. But I use “minimal risk” to refer primarily to a moral threshold. That is, “minimal risk” refers to a moral rule that may inform determinations about when and why the risks of pediatric research are permissible rather than to a particular regulatory concept. Understood in this way, the minimal risk threshold aims to inform as well as unpack current guidance for pediatric research.

3
In some instances these activities may come to benefit the child later in life; in others, benefits may never materialize. Either way, these are instances of risks we may wish to take even without corresponding benefit that are not easily accommodated by the charitable participation interpretation.

4
Robert Nelson suggests a third problem with the charitable participation interpretation. He argues that the root of the problem with this view is that it is motivated by the desire to find an empirical standard for the concept of minimal risk (Nelson 2007, 570). He points out that while empirical data is an important part of the assessment of permissible research risks, the appropriate interpretation of minimal risk should be treated as a value-laden problem. I will return to the idea that an empirically driven threshold is not appropriate in later sections of this paper.

5
This interpretation is endorsed by the National Commission (1976) and in the current U.S. DHHS regulations, in the recommendations of the ad hoc group for the development of implementing guidelines for Directive 2001/20/EC (2008)[Other P-438], and in the Indian Council for Medical Research (2006) (though at times it is combined with the risks of routine examinations threshold).

6
See Binik and Weijer (2014) for an argument for why an unconstrained daily life threshold does not necessarily permit children living in dangerous situations to face higher risks in research without benefit (393–95).

7
At times, this is also understood as referring to a subset of healthy children, such as healthy children in a safe environment.

8
Additional questions for the undue burden argument include further consideration of what is meant by the objective goods, what degree of these goods is sufficiently high, and the implications of this account for current regulations concerning research with children.

9
Wendler also objects to the idea that research risks replace without increasing daily risks (2010, 68). He argues that the risk replacement argument fails because daily activities that are replaced for the sake of research participation are not forgone, but simply delayed until after the trial. For instance, the car trip that was skipped for research participation will simply be delayed until later (2010, 68). He argues that given that both experiences will ultimately be undertaken, the child’s risk exposure will be increased. He then concludes that the risk replacement argument is unsuccessful. This objection is not successful. Proponents of the risk replacement argument are not calculating the aggregative risks a child will face over the course of a day, a week, or during the duration of the clinical trial. The claim is that *at any particular time*, a child will either be undergoing a research procedure and exposed to research risks or the child will be undertaking her usual daily risks. It follows that Wendler’s claim that risks such as a car trip to the mall will simply be delayed until the end of the study does not undermine the risk replacement justification for the risks-of-daily-life threshold.

10
I use group to refer to the nuclear or extended family, but also to broader groups, including neighborhoods, communities, or cities.

11
Additional work on this defense of the risks-of-daily-life will consider how the proposed standard may be implemented in practice. To do so, it will elaborate on the substantive goods of childhood and propose a method in which they may help to guide ethics committees’ determinations about the permissibility of nontherapeutic research procedures. [Other P-439]
